# Stable fixation using absorbable sutures in craniofacial surgery in patients over 24 months of age—a retrospective study

**DOI:** 10.1007/s00381-024-06377-w

**Published:** 2024-04-08

**Authors:** Julian Faber, Christian Linz, Hartmut Böhm, Felix Kunz, Tilmann Schweitzer

**Affiliations:** 1 Department for Oral and Craniomaxillofacial Plastic Surgery, University Hospital Cologne, Kerpener Straße 62, 50937 Cologne, Germany; 2 Department of Oral and Craniomaxillofacial Plastic Surgery, University Hospital Würzburg, Pleicherwall 2, 97070 Würzburg, Germany; 3 Department of Orthodontics, University Hospital Würzburg, Pleicherwall 2, 97070 Würzburg, Germany; 4 Department of Neurosurgery, Section of Pediatric Neurosurgery, University Hospital Würzburg, Josef-Schneider-Straße 2, 97070 Würzburg, Germany

**Keywords:** Fronto-orbital advancement, Craniofacial, Absorbable sutures, Resorbable fixation, Post-operative stability, Cost-effective osteosynthesis

## Abstract

**Purpose:**

In craniofacial surgery, the stable fixation of transposed bone segments is crucial in order to ensure good long-term results. The use of absorbable material in fixation avoids the need for a second surgery, which would otherwise be required to remove osteosynthesis material. The authors of the present manuscript have already demonstrated that absorbable sutures ensure the stable fixation of bone segments in patients up to 24 months of age. However, it has thus far remained unclear whether stable fixation is possible in older patients by using only absorbable sutures due to the slower bone remodelling and prolonged healing time in this cohort.

**Method:**

For the present study, osteosynthesis was performed in 50 patients ranging from 25.7 to 192.1 months of age (mean, 61.4 ± 21.7 months) using solely absorbable sutures (PDS II^®^, Ethicon, Germany). Post-operative stability and possible restrictions—such as foreign body reactions—were evaluated within clinical and radiological routine follow-ups.

**Results:**

All children demonstrated clinically and radiologically stable osteosynthesis both directly post-operatively and in follow-ups. No significant foreign body reaction could be seen.

**Conclusion:**

The present study demonstrates—for the first time—that absorbable sutures with a longer absorption period are also very well suited for the fixation of bone segments in patients over 24 months of age. The sole use of absorbable sutures in children over 24 months of age is a safe procedure with nearly no foreign body reactions. The procedure enables stable and highly cost-effective osteosynthesis without altering the osteotomy design.

## Introduction

Craniofacial malformations can exhibit vastly different manifestations that range from simple monosutural craniosynostosis to complex multisutural syndromal craniosynostosis. In craniofacial surgery, bone segments are mobilised, rearranged, and fixed in a new position. Within this form of surgery, it is crucial that stable osteosynthesis with broad and firm communicating bone edges enable correct ossification and furthermore unimpaired skull growth.

The first materials that were used to fixate bone segments were simple wire ligaments. However, while the development of titan micro-plates resulted in greater stability, other problems remained [1–3]. Aside from foreign body reactions and palpable structures such as wires, plates, and screws, the intracranial transmigration of foreign material due to appositional bone growth in the first years of life has been described [4–7]. Due to the metallic characteristics of these structures, temperature-induced pain and artefacts in radiologic examinations have occurred as well. Additionally, a second surgery is usually necessary to remove the osteosynthesis material [4–8].

In the 1990s, the development of absorbable plates and screws was regarded as an evolutionary milestone in craniofacial surgery. The need for material removal and problems that resulted from the metallic material no longer existed. However, some authors still reported less stability and a long-lasting foreign body reaction due to degradation-resistant polymer crystals [9]. In addition, the loss of tensile strength and difficult handling—including the risk of screw fracture and the need for tapping the screw holes—were described [10, 11]. While the price of absorbable plates and screws is now similar to that of titan micro-plates and screws, these absorbable plates and screws were originally ten times as expensive.

To define the ideal osteosynthesis material for craniofacial surgery in infants, it should (1) ensure stable fixation both for and during the bony healing process, (2) be degradable, and (3) be cost-effective. Due to different timepoints and patient ages of craniofacial surgery, surgeons face diverse, age-dependant bone conditions that differ in thickness, osteosynthetic potential, and mobility. In summary, craniofacial surgeons have to choose the ideal osteosynthesis material based on the age of the patient and the patient’s characteristic bone condition at the time of surgery.

In an earlier study, the present authors presented Vicryl^®^ (Ethicon, Germany) sutures as an ideal material for rigid osteosynthesis in infants less than 24 months of age [12]. However, since bone remodelling in children older than 24 months is slower and less complete compared with in younger children, it remained unclear whether the sole use of absorbable sutures ensures rigid fixation in this special patient cohort [13]. Therefore, in the present study, we investigated PDS II^®^ (Ethicon, Germany) sutures in infants over 24 months of age for the first time.

## Material and methods

In this retrospective study, a total of 50 patients who ranged from 25.7 to 192.1 months of age (mean, 61.4 ± 21.7 months) at the time of surgery were included. The current study was based on a retrospective design using a standard surgical approach without alternating the standard procedure. The only modification was the use of absorbable sutures as osteosynthesis material. As shown by Linz et al., this technique is a standard procedure [12]. Therefore, there was no need to obtain an ethics vote.

The study was carried out according to the Declaration of Helsinki.

All patients suffered from craniosynostosis of at least one cranial suture, and all underwent active remodelling via osteotomy and bone transposition. Osteosynthesis was performed solely by using absorbable sutures (PDS II^®^, in some cases in combination with Vicryl^®^) size 1–0 or 2–0 depending on the presumable tension load. PDS II^®^ was chosen since it offers a long-lasting tensile strength up to 6 weeks and is completely resorbed after half a year [14].

The surgeries took place between 2009 and 2019. At least one of two experienced craniofacial surgeons (T.S. or H.B.) always participated in the surgery.

Surgery was performed in the usual way depending on the affected sutures and the underlying syndrome. The only modification in comparison to described techniques was the use of absorbable sutures as osteosynthesis material.

Initially, we identified 91 patients. Forty-one patients were excluded from the study. Most of the excluded patients were missing post-operative radiologic examinations. In some patients, we had to use absorbable plates to guarantee a stable fixation, because the communicating edges of the newly arranged bone were too short.

Follow-up was performed within the scope of normal post-operative clinical examinations and included routine skull radiographs in a.p. and lateral projection. In very rare cases, for further assessment or a follow-up examination of the intracranial situation in syndromic craniosynostosis, an MRI replaced these X-rays. Follow-up radiographic imaging was analysed for the degree of ossification. Special attention was paid to possible post-operative dislocations that had resulted from a loosening or tear of the applied suture material. Radiographic follow-up took place at 2, 4, 8, and 12 years of age, respectively, or even more frequently in cases with signs of elevated intracranial pressure.

Post-operative stability itself was thoroughly tested via palpation by an experienced neurosurgeon.

Additionally, the wounds were examined for clinical signs of infection or foreign body reaction such as swelling, redness, hyperthermia or systemic reactions.

## Results

Fifty children (33 male, 17 female) with premature synostosis of at least one suture were included in the study. Mean age at initial visit in the craniofacial centre Würzburg was 17.2 months (± 18.0 months; ages between 0 and 84.9 months) (see Table 1). Of these 50 patients, 23 children were diagnosed with a monosutural synostosis. The other 27 children suffered from a combined synostosis of at least two sutures. In 21 children, a syndromic condition was the reason for the synostosis. Seven children suffered from either Crouzon syndrome or Saethre–Chotzen syndrome. In four patients, a pansynostosis in association with neonatal or infantile hypophosphatasia was diagnosed, and in two patients, an Apert syndrome was diagnosed. One patient suffered from CDAGS (craniosynostosis, delayed closure of the fontanelle, anal, genitourinary, and skin abnormalities syndrome) (see Table 2).

**Table 1:** Patient details

**Patient details**	**Value**
Sex, male/female, *n*	33/17
Syndromal, *n*	21
Non-syndromal, *n*	29
Mean age at time of initial visit, *months* (SD)	17.2 (18.0)
Mean age at time of surgery, *months* (SD)	61.4 (21.7)
Mean post-operative hospitalisation, *days* (SD)	7.5 (1.5)
Mean age at last follow-up examination, *months* (SD)	79.2 (25.5)
Surgical and material-related complications, *n*	0

**Table 2:** Synostosis

**Synostosis**	**Number of patients**
Monosutural	23
Sagittal	16
Unilateral coronal	5
Metopic	2
Multisutural (≥ 2)	27

Primary surgery is usually performed during the first 15 months of life [12, 13]. In the present study, however, patients underwent surgery at the age of 24 months or older due to a rather late initial visit in our centre and/or to a recurrence situation after initial surgery. Mean age at the time of surgery was 61.4 months (± 21.7 months; ages between 25.7 and 192.1 months). Mean time of post-operative hospitalisation was 7.5 days (± 1.5 days; range of between 4 and 31 days).

One child suffered from post-operative encephalitis, which resulted in a prolonged post-operative hospitalisation of 31 days due to prolonged antibiotic therapy. However, there was no infection at the site of surgery, nor was there compromised wound healing in the further course of treatment.

Primary fronto-orbital advancement (FOA) was performed in 18 cases, recurrent FOA in five cases, total cranial vault remodelling in 22 cases, and recurrent total cranial vault remodelling in five cases.

**Fig. 1:**
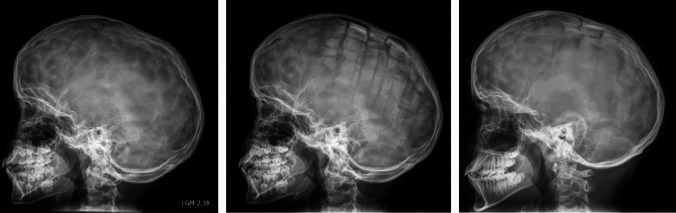
Stable fixation of newly arranged bone segments with PDS sutures. Boy with premature sagittal synostosis, primarily no surgery; skull radiographs as controls revealed digital impressions (left side). Cranial remodelling was performed at 10 years, 5 months (fixation with PDS II^®^). Follow-ups at 5 months post-operatively (middle) and 29 months post-operatively (right) showed no signs of dislocation and demonstrated improving digital impressions

Surgery of a FOA was performed with advancement of a frontal bone segment and an orbital bandeau in a tongue and groove fixation. For total cranial vault remodelling, bone segments were mobilised after coronal osteotomies and refixed after z-shaped osteotomy and newly formed segments (see also Fig. 1).

On average, 30 sutures were used in a FOA. The costs both per unit and for a standard FOA are presented in Table 3.

**Table 3:** Costs of different osteosynthesis materials

Material		Price/unit	Price/FOA (estimated)
Absorbable	*Suture (PDS II*^®^*)*	~ 3 €	~ 90 €
	*Plate (LactoSorb*^®^*)*	~ 50 €	> 1100 €
	*Screw (LactoSorb*^®^*)*	~ 30 €	
Titan	*Plate (Level One Fixation*^®^*)*	~ 45 €	> 1000 €
	*Screw (Level One Fixation*^®^*)*	~ 22 €	

*FOA* fronto-orbital advancement

Mean age at the time of follow-up with radiologic examination was 79.2 months (SD, 25.5 months; 30.5 to 244.2 months).

Clinical examinations during post-operative follow-up were carried out by experienced paediatricians and neurosurgeons and showed no signs of inflammation or foreign body reactions, such as local redness, hyperthermia, or rejection/systemic reactions. Radiologic examinations demonstrated stable osteosynthesis with firm bony union, a preserved tilt angle, and no dislocations at all, as can be seen in Fig. 1.

## Discussion

Following osteotomy, stable fixation of the newly positioned bone arrangement is the decisive step in successful craniofacial surgery. In order to ensure stable fixation, different osteosynthesis materials have been used. Aside from wire ligatures, titan micro-plates were the material of choice until the development of absorbable polymers [15–17].

Although titan micro-plates led to even larger artefacts in radiographic imaging, they were easier to handle and enabled good stability. However, these non-resorbable materials bore several drawbacks, such as a trans-cranial migration in the course of the appositional bone growth of the skull. This trans-osseus migration even led to a final intra-dural position of the originally extra-cranial positioned plate [6]. Additionally, possible bone growth retardation or temperature-induced pain as well as pain and palpable plates and screws have been reported. Another critical element to consider involves the need for a second surgery to remove this material either at a certain point in time or due to the above-mentioned problems [4–7, 18].

In the 1990s, with the development of absorbable plates and screws that consist of biodegradable polymers, most of these problems appeared to be solved. Indeed, there was no longer any need for a second surgery in order to remove the material, no issues arose due to the use of metallics, the biodegradable polymers had a far less negative influence on the skull’s growth, and practically, no trans-osseus migration occurred [19, 20]. This new group of material seemed to be ideal for osteosynthesis; however, new problems soon arose. Aside from the relatively high costs, acute and long-lasting foreign body reactions were described even years after implantation [9, 11, 21–23]. Additionally, in patients with low bone thickness, less stability could be achieved by absorbable material. Moreover, fractures of the osteosynthesis material were also reported [24].

The ideal osteosynthesis material should (1) ensure stability throughout the ossification process, (2) be degradable without foreign body reaction, and (3) therefore not transmigrate through the bone. Additionally, the material should not require major changes in the surgical process. Moreover, the economic element should not be underestimated. By considering all these items, we previously presented a cost-effective and stable yet easy-to-use approach with the application of Vicryl^®^ sutures in children younger than 24 months of age [12].

In children over 24 months of age, re-ossification after surgery is slower and less complete. Compared with in younger children, bone metabolism in children over 24 months is reduced, and wound healing generally takes longer. Therefore, longer stable fixation of the newly arranged segments is needed [13]. Vicryl^®^ is the perfect material in children under 24 months of age because it lacks longer stability due to its degradation time of around only two and a half months [14].

In contrast, PDS II^®^ shows delayed absorption time. As a result, the present study focussed on the sole use of PDS II^®^ sutures—sometimes in combination with Vicryl^®^—as an optimal osteosynthesis material in children over 24 months of age.

Like most absorbable materials, PDS II^®^ is absorbed via hydrolysis. Additionally, PDS II^®^ offers slower absorption and therefore longer maintenance of a stable fixation of up to 6 weeks. According to the manufacturer, around 70% of the tensile strength remains after 2 weeks, and approximately 25% remains 6 weeks after implementation. The absorption process is finally completed after 6 months [14].

In 50 patients, fixation of the newly arranged bone segments was exclusively performed with PDS II^®^ sutures. Based on the localisation, bone thickness, and patient age, the probable tensile load was evaluated intraoperatively by the surgeons. PDS II^®^ 2–0 was usually used. In case of a presumably higher tensile load (e.g. in a recurrence situation), PDS II^®^ 1–0 was applied.

The well-established surgery procedure was not changed. The only difference in technical matter was the use of sutures instead of plates and screws as osteosynthesis material.

Long and closely connected bone edges as well as a meticulous knotting technique are known to be crucial prerequisites for osseous knitting and subsequent sustained bone stability. As a technical note, we set at least four simple knots in PDS II^®^ sutures and left longer thread ends that were bent by the restored galeal flap in order to minimise skin irritation by these more rigid sutures. No special knotting technique is used.

In 2003, Fearon et al. revealed that the use of only absorbable sutures in craniofacial surgery is sufficient for stable fixation. However, the authors changed the design of the osteotomy in order to improve the stability of the newly arranged segments [25]. In 2016, Linz et al. demonstrated that a change in osteotomy design is not necessary when absorbable sutures are used [12]. In the present study, we confirmed these findings.

As mentioned above, an ideal osteosynthesis material should be economic. Compared with absorbable or titan plates and screws, absorbable sutures are much more cost-effective. During a fronto-orbital advancement (FOA), 30 threads are usually knotted in order to ensure stable fixation of the newly arranged bone segments. These threads cost ca. 90 € in total.

In the literature, LactoSorb^®^ (Zimmer Biomet, Germany) has been used as a resorbable plate system [26]. One 25-mm- × -50-mm plate alone costs around 50 €, while a titan micro-plate with four holes—such as Level One Fixation^®^ (KLS Martin, Germany)—costs around 45 €. We conservatively estimated the costs of a FOA using ten titan micro-plates or four 25-mm- × -50-mm LactoSorb^®^ plates and the corresponding screws (see Table 3). As can be seen, the costs are ten to 12 times lower when only absorbable sutures are used.

In the craniofacial centre Würzburg, post-operative follow-up examinations (which usually include plain skull radiographs and MRIs in selected cases when indicated) are routinely performed until the age of 12 for all patients. Although the bony skull might demonstrate some increase in size due to growing bone thickness up to the end of puberty, brain expansion itself—and therefore also the possible discrepancy between required and possible space—ceases by the age of 12 years. Only in syndromic craniofacial malformations or selected cases of later aesthetic corrections were additional radiographs performed. Although these ongoing examinations were not part of the study, no contradictory results were seen in this timeframe.

In all cases, clinical assessment within the scope of post-operative follow-up examinations yielded stable osteosynthesis results. Additionally, skull radiographs in two planes or MRI examinations demonstrated stable bony unions without any misalignment of the translocated segments.

In contrast to absorbable plates and screws, for which foreign body reactions have been described in the literature, we did not observe signs of foreign body reactions during follow-up, even after several months [9, 11, 21–23].

For more than 15 years, the exclusive use of absorbable sutures as osteosynthesis material has been successfully implemented in the craniofacial centre Würzburg. As already demonstrated with Vicryl^®^ as osteosynthesis material in children up to 24 months of age, the present study revealed that stable osteosynthesis with absorbable PDS II^®^ sutures also works in children over 24 months of age [12].

In this study, the oldest child was about 16 years old at the time of surgery. Therefore, no recommendation can be made for the sole use of absorbable sutures for children over 16 years of age or adults with completed bone growth.

## Conclusion

To our knowledge, ours are the first results of stable fixation of bone segments to be made solely with absorbable sutures in children older than 24 months. The method of fixation with absorbable PDS II^®^ [14] alone offers stable and rigid osteosynthesis at extremely low costs in comparison with currently implemented fixation techniques and thus fulfils many of the requirements of ideal osteosynthesis in craniofacial surgery.

## Data Availability

The datasets used and/or analysed during the current study are available from the corresponding author on reasonable request.
